# Supplementary material for: Maintenance of cytoplasmic and membrane densities shapes cellular geometry in Escherichia coli
Source: Nat Commun. 2025 Dec 22;16:11557. doi: 10.1038/s41467-025-67553-3 (PMC12749242; doi:10.1038/s41467-025-67553-3)
Supplement: Supplementary file 1 — Supplementary Information [file 41467_2025_67553_MOESM1_ESM.pdf]

Supplementary Information for "Maintenance of Cytoplasmic and Membrane Densities Shapes Cellular Geometry in *Escherichia coli*"

Griffin Chure[1, *], Roshali T. de Silva[1, 2], Richa Sharma[1], Michael C. Lanz[1,3], and Jonas Cremer[1,4, *]

[1]Department of Biology, Stanford University, Stanford, CA, USA
[2]Current Address: School of Life Sciences, Arizona State University, Tempe, AZ, USA
[3]Chan-Zuckerberg Biohub, San Francisco, CA, USA
[4]Bio-X, Stanford University, Stanford, CA, USA
[*]For correspondence: GC `griffinchure@gmail.com`; JC `jbcremer@stanford.edu`

Contents

I Supplementary Figures **2**

II Supplementary Tables **10**

III Supplementary Notes **11**

1 Strains 11
2 Image Processing 11
3 Bayesian Inference 11
4 Relating Ribosomal Proteome Allocation ϕ_{rib} and the RNA-to-Protein Ratio 14

I Supplementary Figures

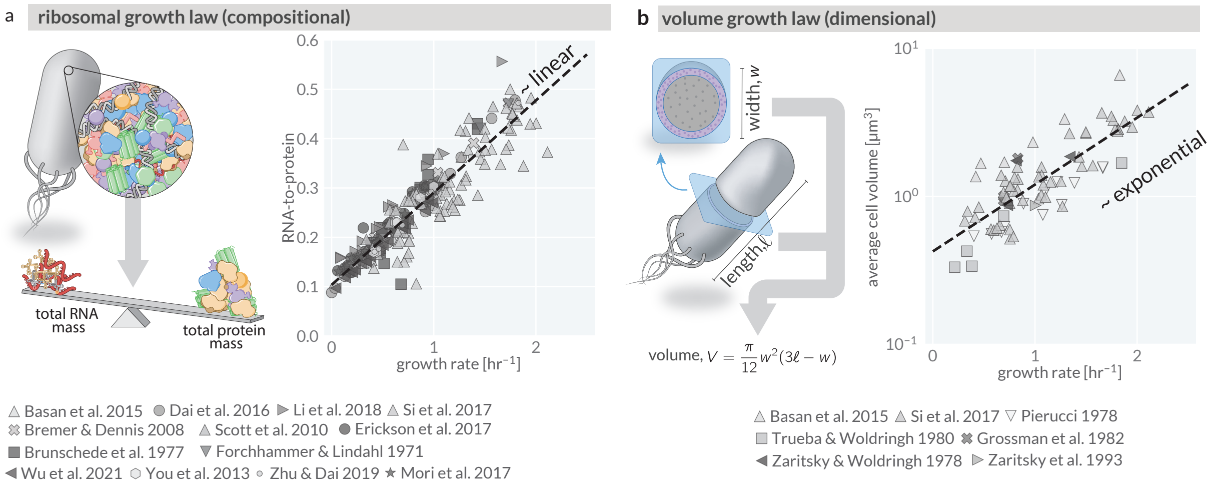

Supplementary Figure 1: The compositional and geometric growth laws of *E. coli*. a The compositional growth law–also termed the "ribosomal growth law"–noting that the bulk RNA-to-protein ratio scales approximately linearly with the steady-state growth rate. **b** The volume growth law noting that *E. coli*'s average cell volume scales approximately exponentially with the bulk growth rate. Dashed lines represent empirically-determined regressions to pooled data sets. Complete references for all data given in the data vvailability statement.

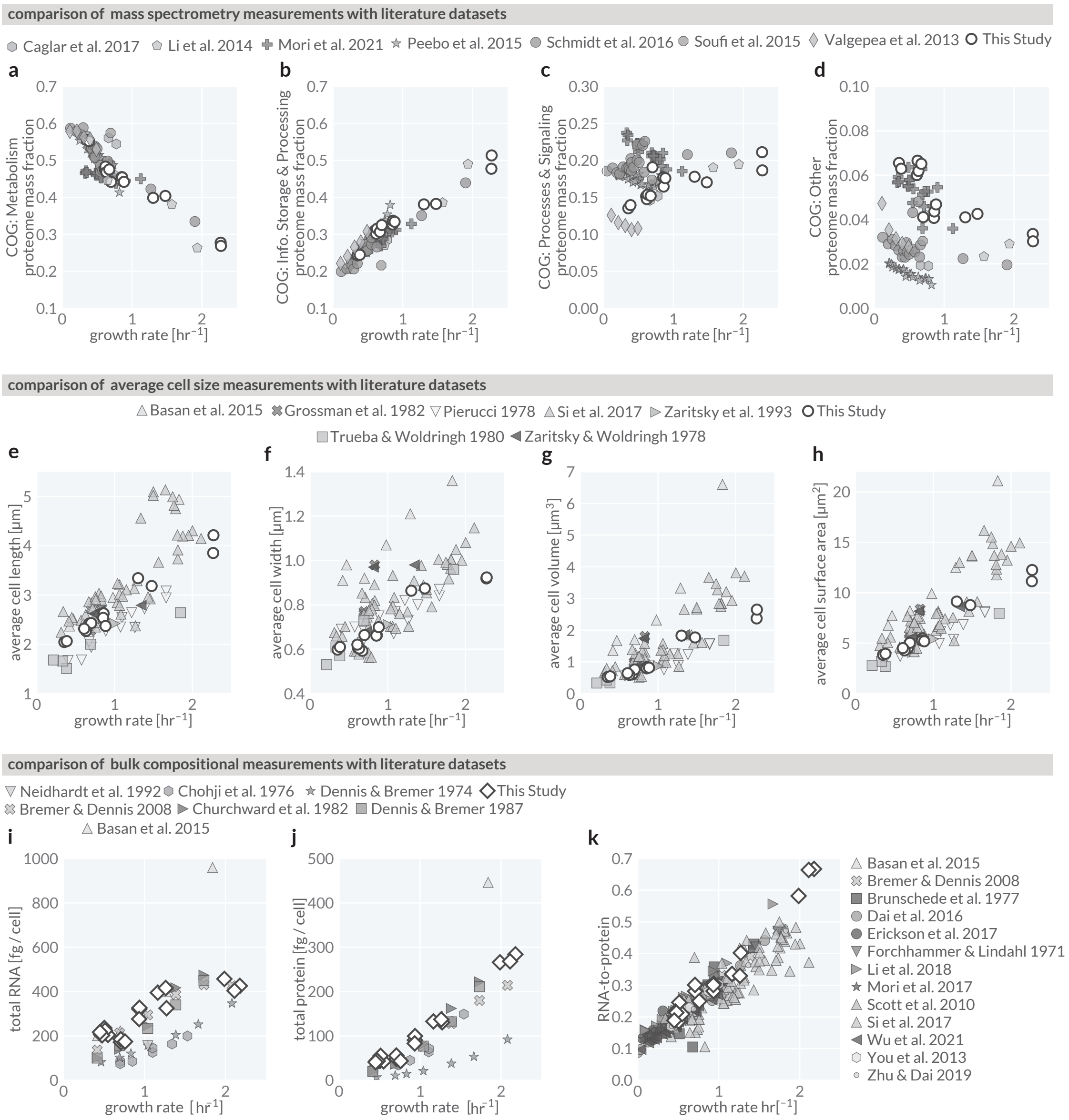

Supplementary Figure 2: Comparison of measurements from this study with those from the literature. (**a-d**) Comparison of mass spectrometry measurements from this study (white-faced circles) with reported literature *E. coli* proteomics data, grouped by Clusters of Orthologous Groups (COG [1]) functional annotation. **a** Metabolism; COG Letters: C, G, E, F, H, I, P, Q. **b** Information storage and processing; COG Letters: J, A, K, L, B. **c** Cellular processes and signaling; COG letters: D, Y, V, T, M, N, Z, W, U, O. **d** Other; COG Letters: R, S. (**e-h**) Comparison of average cell dimensions from this study (white-faced circles) and literature studies (markers). (**i-k**) Comparison of bulk biochemical measurements from this study (white-faced diamonds) with literature measurements. Citations for literature studies are provided in the data availability statement.

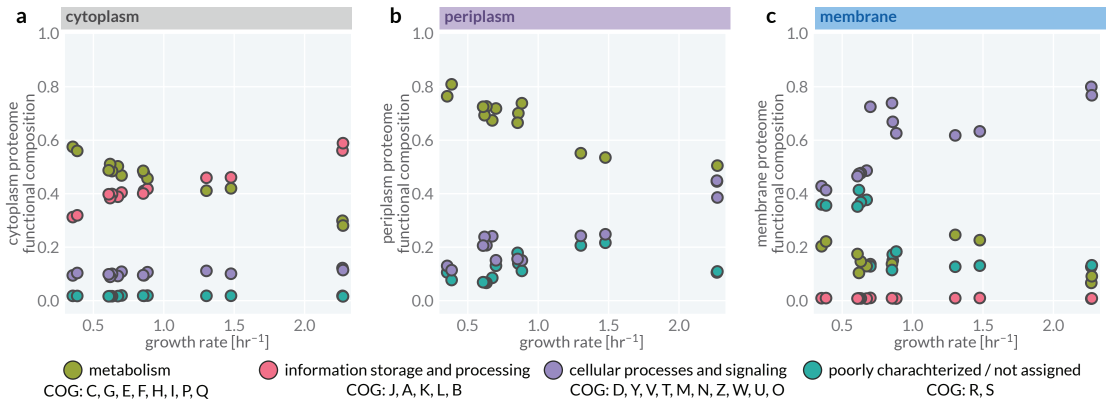

Supplementary Figure 3: Functional composition of proteomics data within different compartments. Total fraction of proteome occupied by each COG category in the **a** cytoplasm, **b** periplasm, and **c** membranes. COG categories are the same as described in Supplementary Fig. 2.

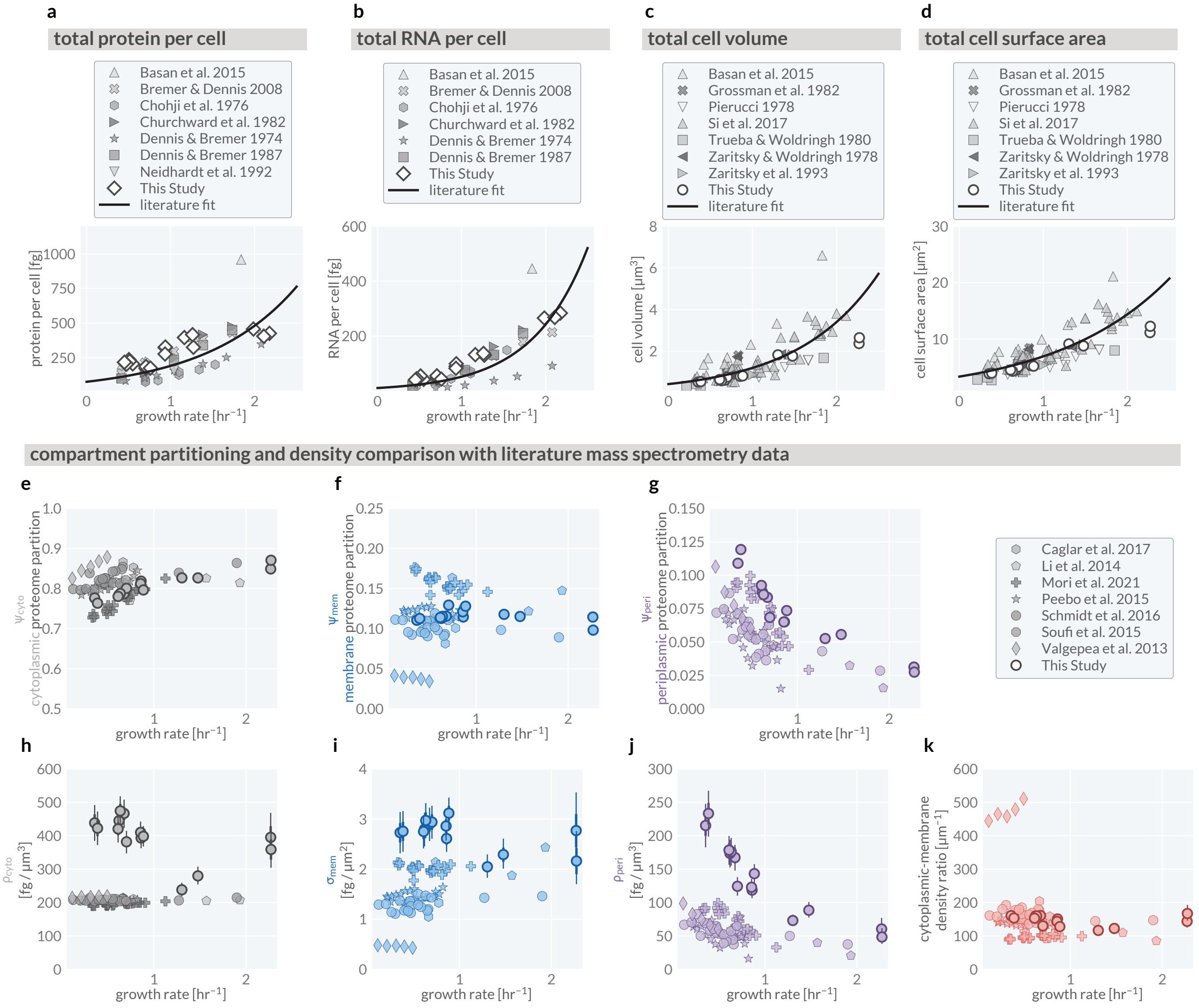

Supplementary Figure 4: Comparison of partitioning coefficients and macromolecular densities with literature data. Literature data for **a** total protein per cell, **b** total RNA per cell, **c** average cell volume, and **d** average cell surface area from literature studies, overlaid with exponential fits. Open-faced glyphs correspond to measurements from this study. Proteome partitioning coefficients for **e** the cytoplasm ψ_{cyto}, **f** membrane ψ_{mem}, and **g** periplasm ψ_{peri} from this study compared with literature data. Inferred macromolecular densities in **h** the cytoplasm ρ_{cyto}, **i** areal protein mass density in the membrane σ_{mem}, and **j** protein density in the periplasm ρ_{peri} from the literature data and fit quantities in (**a**-**d**). dark-colored circles correspond to estimates from our data with thick and thin error bars denoting the bounds of the 95% and 68% percentile bounds of the posterior probability distributions. **k** The empirical cytoplasm-membrane density ratio is shown as the ratio of value in **h** and **i** for each literature data point, respectively.

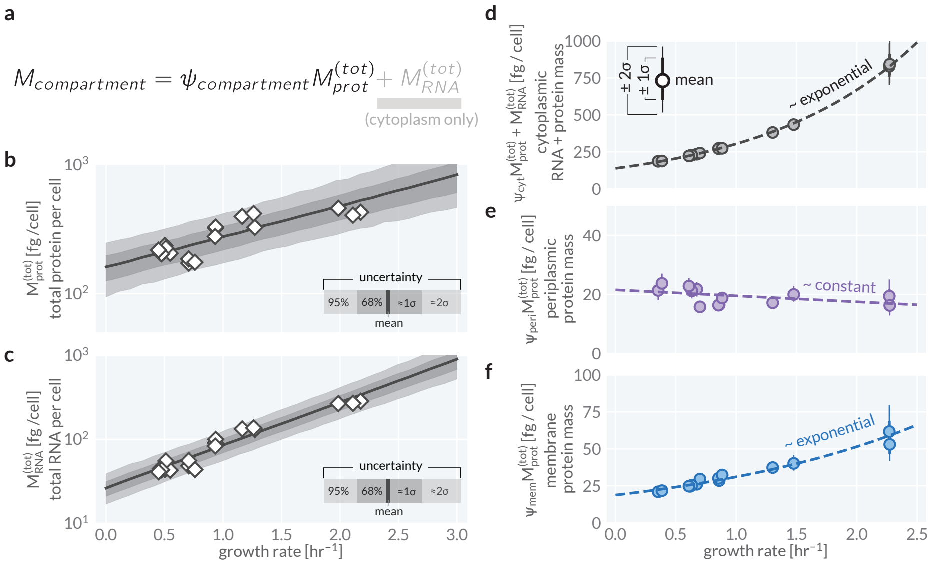

Supplementary Figure 5: Total compartment masses per cell. **a** The mass of a compartment $M_{compartment}$ is defined as the product of the proteome allocation $\psi_{compartment}$ with the total per-cell protein mass $M_{prot}^{(tot)}$. For the cytoplasm, total mass includes the total RNA mass M_{RNA}. **b** Total protein per cell and **c** total RNA per cell measured in bulk for this study are shown as white-diamonds. Shaded regions correspond to the credible regions of the Bayesian statistical model described in Supplementary Note 3. **d** Total RNA + protein mass within the cytoplasm as a function of growth rate. **e** Total protein mass within the periplasm as a function of growth rate. **f** Total protein mass within the membranes as a function of growth rate. Points denote the mean of the posterior probability distribution estimated for each quantity. Thin and thick error bars denote the extent of $\pm$ 2 σ and $\pm$ 1 σ of the posterior distribution. Dashed lines correspond to empirical fits to the posterior means. Cytoplasmic and membrane masses assumed to scale exponentially with the growth rate while the periplasmic mass was assumed to scale linearly.

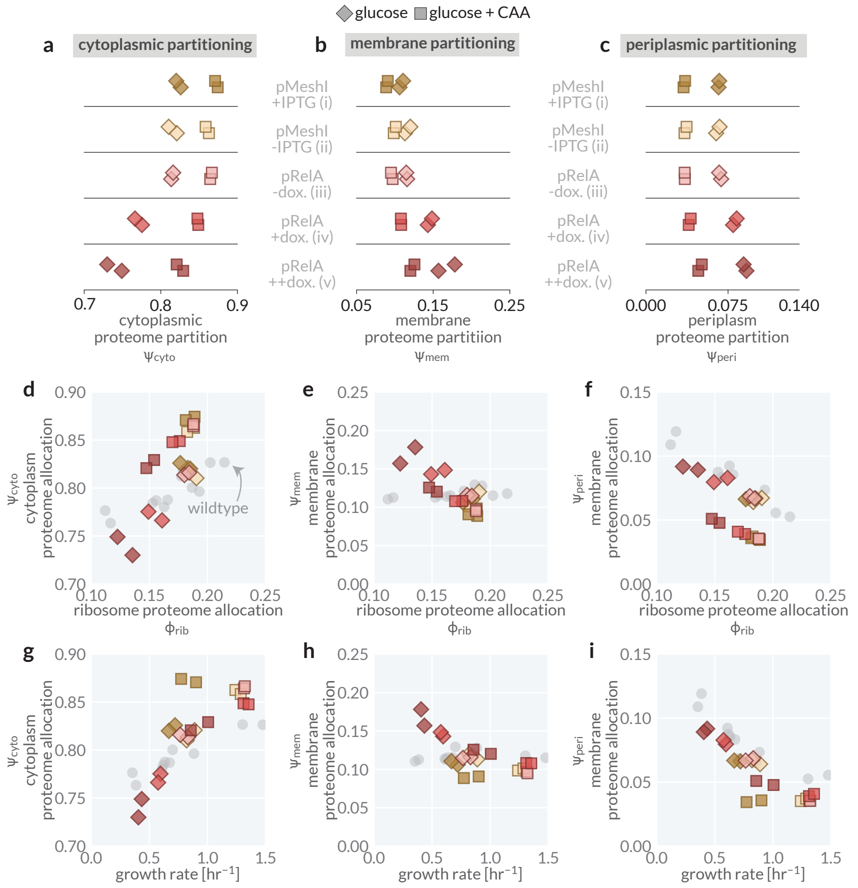

Supplementary Figure 6: Perturbations of intracellular ppGpp and their effects on proteome partitioning between compartments. Observed effects of MeshI or RelA overexpression on **a** cytoplasmic partitioning ψ_{cyto}, **b** membrane partitioning ψ_{mem}, and **c** periplasmic partitioning ψ_{peri} in glucose (diamonds) or casamino acid supplemented glucose minimal medium. Individual points denote biological replicates. The partitioning coefficients are plotted as a function of the (**d-f**) ribosomal proteome allocation ϕ_{rib} and (**g - i**) the steady-state bulk growth rate. Gray circles denote wildtype samples grown in different conditions. MeshI was induced with IPTG (- : 0 µM + : 100 µM,) and RelA was induced with doxycyline (- : 0 ng/mL; + : 2 ng/mL, ++ : 4 ng/mL).

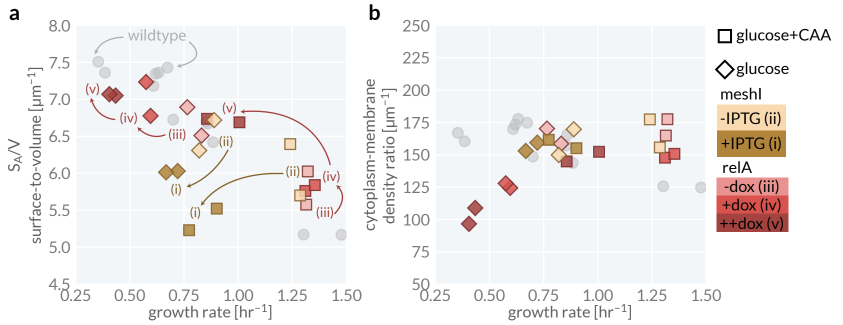

Supplementary Figure 7: Changes in S_A/V and the cytoplasm-membrane density ratio under perturbation of intracellular ppGpp concentrations a Perturbations of intracellular ppGpp concentrations break the dependence of S_A/V on growth rate. The measured surface-to-volume ratio S_A/V is plotted as a function of the steady-state growth rate. MeshI induction in glucose (diamonds) and glucose+CAA (squares) is denoted as trajectory $(ii) \rightarrow (i)$ while RelA induction in each condition is denoted as trajectory $(iii) \rightarrow (iv) \rightarrow (v)$, following the notation shown in Fig. 4. Induction of MeshI (dark gold) decreases both the growth rate and the S_A/V strongly deviating from the wildtype trend. **b** Empirically determined cytoplasm-membrane density ratio for ppGpp perturbation experiments as calculated in Eq. 8. Values for ppGpp perturbation strains are shown in colors and were calculated as described in Methods. MeshI was induced with IPTG ($-$: 0 µM $+$: 100 µM) and RelA was induced with doxycycline ($-$: 0 ng/mL; $+$: 2 ng/mL, $++$: 4 ng/mL).

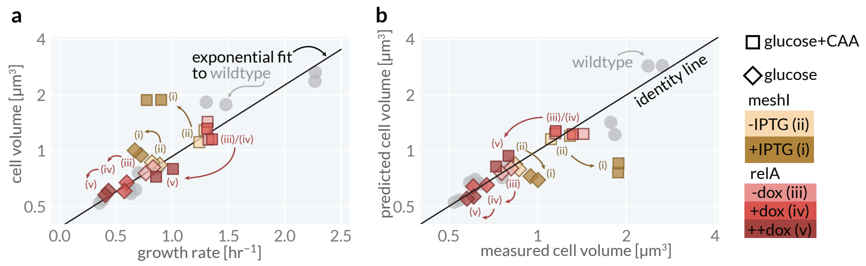

Supplementary Figure 8: Change in cell volume under perturbation of intracellular ppGpp concentration. **a** Measured cell volume as a function of the bulk rate for wildtype (grey circles) and ppGpp perturbations. Black line indicates an exponential fit to the wildtype measurements. **b** The predicted cell volume from the exponential fit shown in a as a function of the measured cell volume under different ppGpp perturbations.

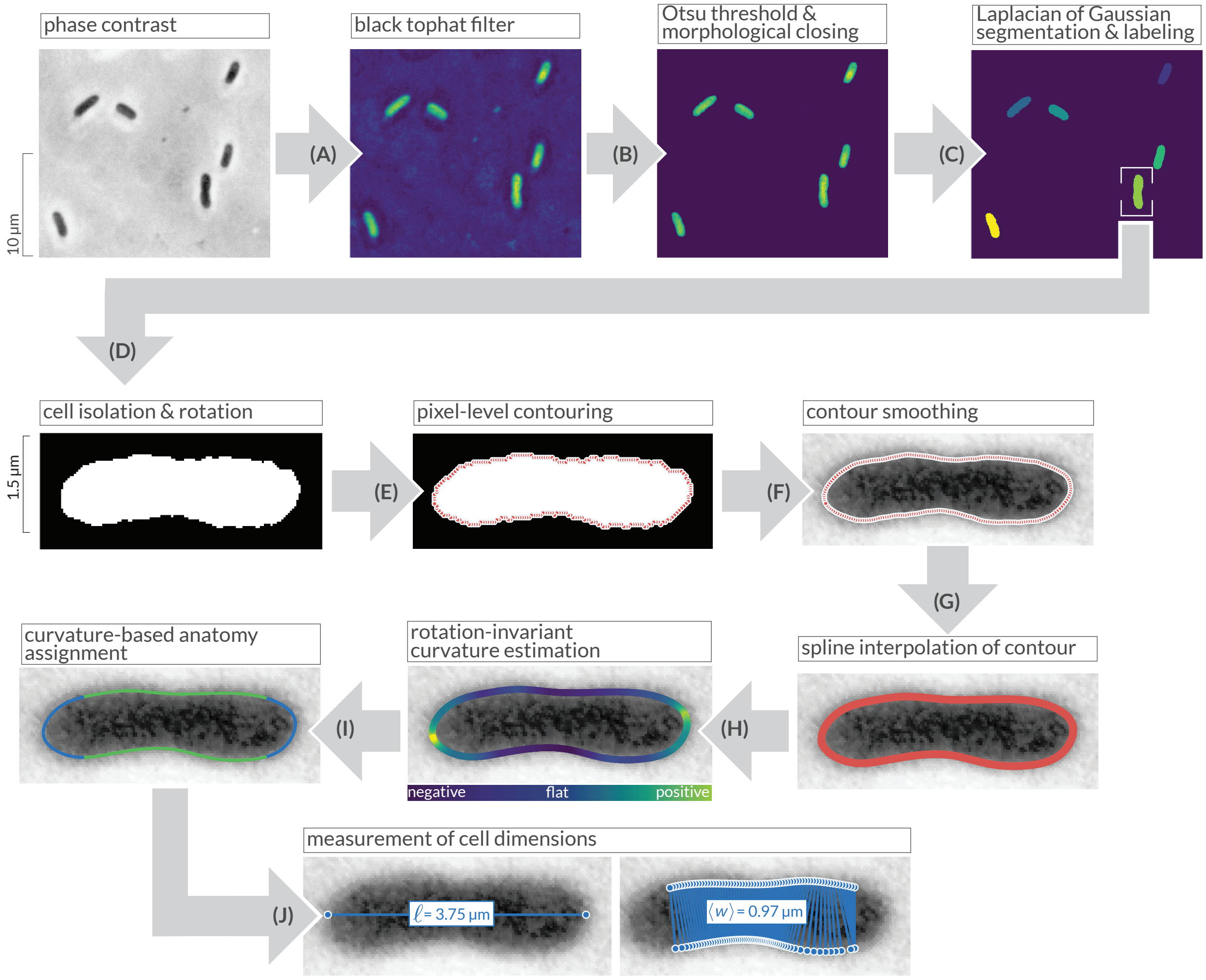

Supplementary Figure 9: Segmentation and cell measurement pipeline. Raw phase-contrast images of cells are passed through (arrow A) a black tophat filter and (B) Otsu thresholding to arrive at emphasized cell boundaries. (C) Laplacian of Gaussian segmentation is then applied to arrive at rough cell masks. For each cell, the sgementation mas is (D) rotated and aligned and the periphery is identified using (E) contouring and (F) Savgol filtering. The isolated contours are then smoothed with (G) spline interpolation. In a clock-wise direction, the (H) curvature of the contour is calculated and used to (I) identify sides and caps. With morphology assigned, cell dimensions are then (J) measured.

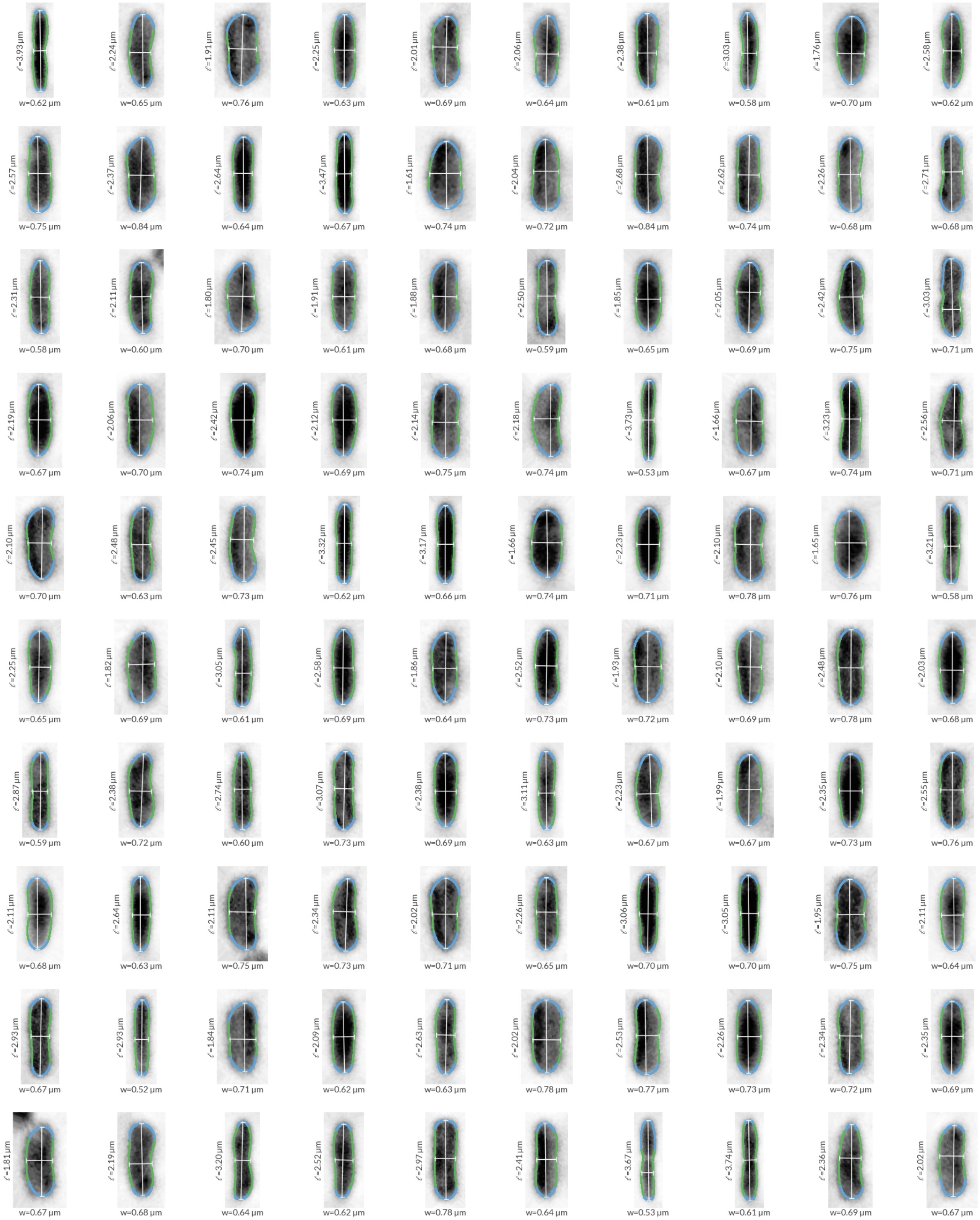

Supplementary Figure 10: Example segmentation of *E. coli* cells grown on a glucose minimal medium to steady-state. Blue and green regions of the contours correspond to the caps and sides of the cells, respectively.

II Supplementary Tables

Supplementary Table 1: Linear regression parameters determined by optimization for key empirical relationships. Values of slope and intercept relative to the mean value of the quantity are given in parentheses. For exponential fits, constant C_0 with unity value of the quantity dimension is included in the log transformation to avoid taking the logarithm of a dimensionful quantity.

Dep. Variable [units]	Indep. Variable [units]	Figure Panel	Slope [units]	Intercept [units]
ψ_{cyto}	λ [hr^{-1}]	Fig. 1b	0.046 [hr]<br>(0.057 [hr])	0.760<br>(0.944)
ψ_{peri}	λ [hr^{-1}]	Fig. 1c	-0.039 [hr]<br>(-0.534 [hr])	0.111<br>(1.53)
ψ_{mem}	λ [hr^{-1}]	Fig. 1d	-0.004 [hr]<br>(-0.038 [hr])	0.120<br>(1.04)
$\psi_{cyto} + \psi_{mem}$	λ [hr^{-1}]	Fig. 1f	0.007 [hr]<br>(0.008 [hr])	0.871<br>(0.992)
ρ_{cyto} [fg/µm^3]	λ [hr^{-1}]	Fig. 2a	-54.3 [fg· hr / µm^3]<br>(-0.138 [hr])	448 [fg / µm^3]<br>(1.137)
σ_{mem} [fg / µm^2]	λ [hr^{-1}]	Fig. 2b	-0.277 [fg · hr / µm^2]<br>(-0.103 [hr])	2.968 [fg / µm^2]<br>(1.10)
$\log_{10}(\rho_{peri}/C_0)$	λ [hr^{-1}]	Fig. 2c	-0.733 [hr]<br>(-0.005)	5.554<br>(0.040)
ρ_{cyto}/σ_{mem} [µm^{-1}]	λ [hr^{-1}]	Fig. 2d	-4.33 [hr / µm^{-1}]<br>(-0.030 [hr])	150 [µm^{-1}]<br>(1.03)
$\log_{10}(M_{prot}^{(cyto)} + M_{RNA}^{(tot)}/C_0)$	λ [hr^{-1}]	Sup. Fig. 5d	0.785 [hr]<br>(0.002 [hr])	4.99<br>(0.014)
$M_{prot}^{(peri)}$ [fg / cell]	λ [hr^{-1}]	Sup. Fig. 5e	-2.135 [fg·hr / cell]<br>(-0.117 [hr])	20.3<br>(1.12)
$\log_{10}(M_{prot}^{(mem)}/C_0)$	λ [hr^{-1}]	Sup. Fig. 5f	0.504 [hr]<br>(0.015 [hr])	2.93<br>(0.090)

Supplementary Table 2: Strains used in this study.

Strain	Source	Description
NCM3722	Laboratory of Terence Hwa	WT
GE463	Plasmid from Addgene, AddGeneID:175594	pMeshI
GE462	Plasmid from Addgene, AddGeneID:175595	pRelA

III Supplementary Notes

1 Strains

All *Escherichia coli* strains used in this work are described in the Supplementary Table 2. Plasmids with MeshI (GE463) or RelA (GE462) under inducible control were acquired from Addgene and transformed into our wildtype NCM3722 host strain.

2 Image Processing

In this work, we directly measured cell size parameters from phase-contrast microscopy images at 100X magnification. We employed an in-house image processing Python pipeline to segment and measure per-cell parameters. The steps of this segmentation algorithm are outlined in Supplementary Notes Fig. 9. Briefly, individual cells are identified in an image through several rounds of filtering and thresholding. Once cell masks are identified, each is rotated and aligned to a common axis and contours are determined by edge detection, smoothing, and spline interpolation. With a spline interpolation in place, the curvature k along the contour along an xy coordinate system at each point is calculated via

$$k = \frac{x'y'' - y'x''}{(x'^2 + y'^2)^{3/2}} \tag{1}$$

Note that this preserves sign of the curvature. By rotating each cell mask to have the same axis of orientation, we can ensure that the sign of the curvature is consistent between individual cells and images.

With an estimate of the cell curvature in place at each position along the contour, we apply a threshold on this value to identify what portions of the contour correspond to the cell sides ($k \approx 0$) versus the cell caps ($|k| > 0$). We identified the cap regions of the cells as the contour points with radii of curvature greater than 0.5 µm. To compute the cell length dimension, we took the length to be the maximum y coordinate difference between contour points of the two caps. For the width, we computed the average minimum pairwise distance between the two cell side contours. Representative segmented cell peripheries are shown in Supplementary Notes Fig. 10 for 100 cells from a single wildtype sample grown in a glucose minimal medium. We found that our segmentation protocols yielded measurements that were in line with literature cell size measurements and exhibited the same apparent growth-rate dependence, as shown in Supplementary Fig. 2(e-h).

3 Bayesian Inference

In this work, we utilize Bayesian statistical methods to systematically propagate all uncertainty from measurements and our model assumptions to generate the final predictions and experimental data presented in this work. In the subsections that follow, we present detailed descriptions of the various components of the inference. In all cases, inferences using literature data and inferences using our suite of experimental measurements were performed independently. Further, we note that *all* inference of model parameters and calculation of estimated membrane protein densities were conducted simultaneously, resulting in a rather large posterior probability distribution that precludes complete enumeration here. However, we invite the reader to examine the full statistical inference models, which are written using the Stan probabilistic programming

language [2], and which are available on the paper GitHub repository (`github.com/cremerlab/density_maintenance`). We find that these models, coupled with the inline comments, are easier to parse than a full mathematical statement.

Speaking generally, we sought to inference the probability of a parameter θ taking on a given value, conditioned on an experimental measurement y. This quantity, termed the *posterior probability distribution* $g(\theta \,|\, y)$ (hereafter called the *posterior*) can be computed using Bayes' rule,

$$g(\theta \,|\, y) = \frac{f(y \,|\, \theta) g(\theta)}{f(y)} \tag{2}$$

where g and f denote probability density functions over parameters and data, respectively. In computing the posterior, one must minimally enumerate the likelihood function $f(y \,|\, \theta)$ and the prior $g(\theta)$. The denominator of Eq. 2 is termed the *evidence* or the *marginalized likelihood* and represents the probability of observing a datum y irrespective of the model. For our purposes, we can treat this as a normalization constant and neglect it. As such, Eq. 2 becomes

$$g(\theta \,|\, y) \propto f(y \,|\, \theta) g(\theta). \tag{3}$$

The likelihood function $f(y \,|\, \theta)$ represents the probability of observing a datum y *given* a particular value of the parameter θ. In all cases for this work, we took the likelihood to have the form of a Gaussian distribution parameterized by a mean μ and homoscedastic error σ,

$$f(y \,|\, \theta) = \frac{1}{\sqrt{2\pi\sigma^2}} \exp\left[-\frac{(y-\mu)^2}{2\sigma^2}\right] \Rightarrow f(y \,|\, \theta) \sim \text{Normal}(\mu, \sigma) \tag{4}$$

where we have introduced a short-hand notation. Asserting a Gaussian likelihood makes an assumption about how the measurements are distributed about a mean value μ, but not *what* that mean value is. The remaining sections of this Supplementary Note outline how we determine what the mean value of this likelihood function is for a variety of components of our models.

Finally, we must also provide a definition of the prior distribution over the parameter $g(\theta)$. This distribution encapsulates all knowledge we have of what the true parameter value of θ might be *without* taking the observations into account. This is a critically important point and each prior choice represents the assumptions and domain expertise we employ in crafting these models. In the subsections that follow, we outline the inferential models and provide our prior choices. We used the Probability Distribution Explorer tool (`distribution-explorer.github.io`) to interactively identify parameter values that yielded prior distributions consistent with our domain knowledge.

To compute credible regions of model predictions and fits from the posterior distributions (such as the uncertainty bands in Fig. 3), we employed Markov Chain Monte Carlo (MCMC) sampling techniques implemented through Stan's probabilistic programming framework [2]. For a given model with a Gaussian likelihood as in Eq. 4, we first draw samples of μ and σ from their posterior distributions. Using these posterior parameter samples, we generate posterior predictive datasets $\tilde{y}$ by drawing from the likelihood $f(\tilde{y} \,|\, \theta) \sim \text{Normal}(\mu_{posterior}, \sigma_{posterior})$. We construct credible intervals at the 68% and 95% levels, which approximately correspond to one and two standard deviations in a normal distribution, respectively. This is achieved by finding the appropriate quantiles of the posterior samples for each parameter of interest. The

process allows us to construct marginal and joint credible intervals, while simultaneously enabling model validation through posterior predictive checks. By comparing the statistical properties of these simulated datasets $\tilde{y}$ to our observed data y, we can diagnose potential model misspecifications, assess the model's predictive performance, and quantify the uncertainty inherent in our parameter estimates.

Inferring the Total Protein and RNA Mass per Cell

In this work, we combined independent measurements of the total protein and RNA per cell in wildtype *E. coli* with our mass spectrometry measurements to calculate various properties, including the total protein masses in each cellular compartment and the corresponding densities.

We assumed that the total protein per cell $M_{prot}^{(tot)}$ scaled exponentially with the steady-state growth rate λ,

$$M_{prot}^{(tot)}(\lambda) = \beta_{prot,0} \exp\left(\beta_{prot,1}\lambda\right), \tag{5}$$

where $\beta_{prot,0}$ and $\beta_{prot,1}$ are phenomenological coefficients. Setting this equation as the mean of a Normal likelihood, we inferred the values of these parameters using MCMC setting the following priors:

$$\beta_{prot,0} \sim \text{Inv.Gamma}(\alpha = 13.21, \beta = 1062)\,;\beta_{prot,1} \sim \text{Normal}(0,1), \tag{6}$$

where Inv.Gamma denotes the Inverse Gamma function. Values for the location and scale parameters of this distribution (α and β) were chosen such that the upper and lower bounds of the 95th percentile were 50 fg/cell and 150 fg/cell, respectively.

We also assumed that the total RNA per cell $M_{RNA}^{(tot)}$ scaled exponentially with the steady-state growth rate λ,

$$M_{RNA}^{(tot)}(\lambda) = \beta_{RNA,0} \exp\left(\beta_{RNA,1}\lambda\right), \tag{7}$$

where $\beta_{RNA,0}$ and $\beta_{RNA,1}$ are the phenomenological coefficients we seek to infer. For this inference, we set the following priors:

$$\beta_{RNA,0} \sim \text{Inv.Gamma}(\alpha = 3.358, \beta = 77.87)\,;\beta_{RNA,1} \sim \text{Normal}(0,1), \tag{8}$$

where Inv.Gamma denotes the Inverse Gamma function. Values for the location and scale parameters of this distribution (α and β) were chosen such that the upper and lower bounds of the 95th percentile were 10 fg/cell and 100 fg/cell, respectively.

Inferring Model Parameters

As derived in the main text, the central prediction of the density maintenance model is that the cellular surface-to-volume ratio S_A/V is related to the cellular composition and proteome partitioning between compartments via

$$S_A/V = \frac{\kappa\psi_{mem}}{2\left[1 - \psi_{mem} - \psi_{peri} + \beta\phi_{rib}\right]} \tag{9}$$

where ψ_{mem} is the proteome partitioning towards the membrane, ψ_{peri} is the partitioning toward the periplasm, κ is the cytoplasm-membrane density ratio, and ϕ_{rib} is the ribosomal proteome allocation, related to the

RNA-to-protein ratio via the proportionality constant β, derived in the next section. To test our model, we sought to estimate the value of κ that could describe how the S_A/V scaled as ϕ_{rib} was varied, requiring us to also estimate how ψ_{mem} and ψ_{peri} depended on ϕ_{rib}.

There are many different functional forms one could use to describe how the ψ's depended on ϕ_{rib}. We made the empirical assumption that the membrane proteome partitioning ψ_{mem} was linearly dependent on ϕ_{rib} with the form

$$\psi_{mem}(\phi_{rib}) = \beta_{0,\psi_{mem}} + \beta_{1,\psi_{mem}}\phi_{rib} \tag{10}$$

where $\beta_{0,\psi_{mem}}$ and $\beta_{1,\psi_{mem}}$ are phenomenological constants we sought to infer. For this inference, we defined the priors to be

$$\beta_{0,\psi_{mem}} \sim \text{Beta}(\alpha = 1.262, \beta = 5.967)\,;\, \beta_{\psi_{mem}} \sim \text{Normal}(0, 1), \tag{11}$$

where Beta denotes the Beta distribution, which is defined on the interval [0, 1]. Values for the location α and scale β parameters of the Beta distribution were chosen such that the lower and upper bounds of the 95th percentile were 0.01 and 0.5, respectively.

For the periplasmic partitioning, we empirically modeled an exponential dependence on ϕ_{rib}, having the form

$$\psi_{peri}(\phi_{rib}) = \beta_{0,\psi_{peri}} \exp\left(\beta_{1,\psi_{peri}}\phi_{rib}\right), \tag{12}$$

where the priors for the phenomenological constants $\beta_{0,\psi_{peri}}$ and $\beta_{1,\psi_{peri}}$ were similarly defined to be

$$\beta_{0,\psi_{peri}} \sim \text{Beta}(\alpha = 1.262, \beta = 5.967)\,;\, \beta_{1,\psi_{peri}} \sim \text{Normal}(0, 10), \tag{13}$$

where a broader prior on $\beta_{1,\psi_{peri}}$ was defined to permit a stronger dependence on ϕ_{rib}. Bounds for α and β in the Beta distribution were empirically chosen such that the lower and upper bounds of the 95th percentile were 0.01 and 0.5, respectively

These dependencies were inferred simultaneously with our inference of κ, given our direct measurement of S_A/V and ϕ_{rib} given Eq. 9. For this inference, we assigned a prior distribution over κ to be

$$\kappa \sim \text{Inv.Gamma}(\alpha = 2.863, \beta = 140.1), \tag{14}$$

where the values for the location α and scale β parameters were chosen such that the lower and upper bounds of the 95th percentile were 20 µm^{-1} and 250 µm^{-1}, respectively.

4 Relating Ribosomal Proteome Allocation ϕ_{rib} and the RNA-to-Protein Ratio

In the main text, we make the assertion that the ribosomal proteome fraction ϕ_{rib} is related to the RNA-to-protein ratio $M_{RNA}^{(tot)}/M_{prot}^{(tot)}$ via the proportionality constant β. This constant can be estimated directly [3,4] given our knowledge of the RNA and protein content of the ribosome [3,5].

The vast majority of total cellular RNA is ribosomal [3], allowing us to state

$$M_{rRNA}^{(tot)} \approx \alpha M_{RNA}^{(tot)}, \tag{15}$$

where $M_{rRNA}^{(tot)}$ is the total rRNA mass of the cell, $M_{RNA}^{(tot)}$ is the total RNA mass and α is a fractional quantity between 0 and 1. We can make the assumption that approximately all rRNA is associated with ribosomal

particles, allowing us to calculate this mass M_{rRNA} as

$$M_{rRNA}^{(tot)} = m_{rRNA} N_{rib}, \tag{16}$$

where m_{rRNA} is the total mass of rRNA associated with a single ribosome and N_{rib} is the total number of ribosomes per cell. Assuming that all ribosomal proteins are within assembled ribosomes, the total number of ribosomes N_{rib} can be calculated from the ribosomal allocation ϕ_{rib} and the total protein mass $M_{prot}^{(tot)}$ as

$$N_{rib} = \frac{\phi_{rib} M_{prot}^{(tot)}}{m_{rib}}, \tag{17}$$

where m_{rib} is the total proteinaceous mass of a single ribosome.

We can combine Eq. 15 - Eq. 17 and solve for the total RNA-to-protein ratio $M_{RNA}^{(tot)} / M_{prot}^{(tot)}$ to yield

$$\frac{M_{RNA}^{(tot)}}{M_{prot}^{(tot)}} = \frac{m_{rRNA}}{\alpha m_{rib}} \phi_{rib} = \beta \phi_{rib}, \tag{18}$$

where β is the proportionality constant relating ribosomal allocation and the RNA-to-protein ratio. Using our knowledge of the total length of rRNA ($\approx$ 4500 nt) and total size of ribosomal proteins ($\approx$ 7500 AA [4]), the precise value of β can be calculated as

$$\beta = \overbrace{\left[\frac{4500\,\text{RNA nt}}{\text{ribosome}} \cdot \frac{340\,\text{Da}}{\text{RNA nt}}\right]}^{m_{rRNA}} \cdot \underbrace{\left[\frac{\text{ribosome}}{7500\,\text{AA}} \cdot \frac{\text{AA}}{110\,\text{Da}}\right]}_{m_{rib}} \cdot \underbrace{\left[\frac{1}{0.85}\right]}_{\alpha} \approx 2.18, \tag{19}$$

which is in close alignment with other calculations [3, 4].

References

[1] Michael Y. Galperin, Yuri I. Wolf, Kira S. Makarova, Roberto Vera Alvarez, David Landsman, and Eugene V. Koonin. COG database update: focus on microbial diversity, model organisms, and widespread pathogens. *Nucleic Acids Research*, 49(D1):D274–D281, January 2021.

[2] Bob Carpenter, Andrew Gelman, Matthew D. Hoffman, Daniel Lee, Ben Goodrich, Michael Betancourt, Marcus Brubaker, Jiqiang Guo, Peter Li, and Allen Riddell. Stan: A probabilistic programming language. *Journal of Statistical Software*, 76(1):1–32, January 2017.

[3] Matthew Scott, Carl W. Gunderson, Eduard M. Mateescu, Zhongge Zhang, and Terence Hwa. Interdependence of Cell Growth and Gene Expression: Origins and Consequences. *Science*, 330(6007):1099–1102, November 2010. tex.ids: scott2010a Publisher: American Association for the Advancement of Science Section: Report.

[4] Griffin Chure and Jonas Cremer. An optimal regulation of fluxes dictates microbial growth in and out of steady state. *eLife*, 12:e84878, March 2023. Publisher: eLife Sciences Publications, Ltd.

[5] Hans Bremer and Patrick P. Dennis. Modulation of Chemical Composition and Other Parameters of the Cell at Different Exponential Growth Rates. *EcoSal Plus*, 3(1), October 2008. Publisher: American Society for Microbiology.
