# Supplementary material for: Maintenance of cytoplasmic and membrane densities shapes cellular geometry in Escherichia coli
Source: Nat Commun. 2025 Dec 22;16:11557. doi: 10.1038/s41467-025-67553-3 (PMC12749242; doi:10.1038/s41467-025-67553-3)
Supplement: Supplementary file 2 — Reporting Summary [file 41467_2025_67553_MOESM2_ESM.pdf]

nature portfolio

Corresponding author(s): Jonas Cremer and Griffin Chure

Last updated by author(s): 11/25/2025

Reporting Summary

Nature Portfolio wishes to improve the reproducibility of the work that we publish. This form provides structure for consistency and transparency in reporting. For further information on Nature Portfolio policies, see our Editorial Policies and the Editorial Policy Checklist.

Statistics

For all statistical analyses, confirm that the following items are present in the figure legend, table legend, main text, or Methods section.

n/a	Confirmed	
☐	☒	The exact sample size (*n*) for each experimental group/condition, given as a discrete number and unit of measurement
☐	☒	A statement on whether measurements were taken from distinct samples or whether the same sample was measured repeatedly
☐	☒	The statistical test(s) used AND whether they are one- or two-sided<br>*Only common tests should be described solely by name; describe more complex techniques in the Methods section.*
☒	☐	A description of all covariates tested
☒	☐	A description of any assumptions or corrections, such as tests of normality and adjustment for multiple comparisons
☐	☒	A full description of the statistical parameters including central tendency (e.g. means) or other basic estimates (e.g. regression coefficient) AND variation (e.g. standard deviation) or associated estimates of uncertainty (e.g. confidence intervals)
☒	☐	For null hypothesis testing, the test statistic (e.g. *F*, *t*, *r*) with confidence intervals, effect sizes, degrees of freedom and *P* value noted<br>*Give P values as exact values whenever suitable.*
☐	☒	For Bayesian analysis, information on the choice of priors and Markov chain Monte Carlo settings
☒	☐	For hierarchical and complex designs, identification of the appropriate level for tests and full reporting of outcomes
☒	☐	Estimates of effect sizes (e.g. Cohen's *d*, Pearson's *r*), indicating how they were calculated

Our web collection on statistics for biologists contains articles on many of the points above.

Software and code

Policy information about availability of computer code

Data collection	Mass spectrometry data was collected and processed via MaxQuant (v2). Details are described in the Methods section.
Data analysis	For data and statistical analyses, we used Python and Stan base code that we customized for this work. All Python code, Stan probabilistic models, and processed data sets are available on the paper's GitHub repository DOI:10.5281/zenodo.10048570accessible.

For manuscripts utilizing custom algorithms or software that are central to the research but not yet described in published literature, software must be made available to editors and reviewers. We strongly encourage code deposition in a community repository (e.g. GitHub). See the Nature Portfolio guidelines for submitting code & software for further information.

Data

Policy information about availability of data

All manuscripts must include a data availability statement. This statement should provide the following information, where applicable:

- Accession codes, unique identifiers, or web links for publicly available datasets
- A description of any restrictions on data availability
- For clinical datasets or third party data, please ensure that the statement adheres to our policy

Raw microscopy images are available to download from the Stanford Data
Repository accessible via DOI: 10.25740/ws785mz0287. All other experimental data are available on the paper's GitHub repository DOI: 10.5281/zenodo.10048570
accessible via

https://github.com/cremerlab/density_maintenance. In this work, we also integrated a large collection of datasets from the literature to evaluate the consistency of our measurements and guide our analysis. The datasets used from these studies are provided in the repository, and the corresponding references are listed in the maintext.

Research involving human participants, their data, or biological material

Policy information about studies with human participants or human data. See also policy information about sex, gender (identity/presentation), and sexual orientation and race, ethnicity and racism.

Reporting on sex and gender	Does not apply.
Reporting on race, ethnicity, or other socially relevant groupings	Does not apply.
Population characteristics	Does not apply.
Recruitment	Does not apply.
Ethics oversight	Does not apply.

Note that full information on the approval of the study protocol must also be provided in the manuscript.

Field-specific reporting

Please select the one below that is the best fit for your research. If you are not sure, read the appropriate sections before making your selection.

☒ Life sciences ☐ Behavioural & social sciences ☐ Ecological, evolutionary & environmental sciences

For a reference copy of the document with all sections, see nature.com/documents/nr-reporting-summary-flat.pdf

Life sciences study design

All studies must disclose on these points even when the disclosure is negative.

Sample size	Measured quantities like growth rates or proteome fractions are highly reporducable. Generally, a sample size of 2-3 was thus chosen as common in the field.
Data exclusions	No data was excluded.
Replication	2 to 3 biological replicates were generally taken. High similarities of these measurements confirm reprducability.
Randomization	Does not apply, same cell types have been used for culture experiments in different growth conditions.
Blinding	Cultures from different cell types and growth conditions have been analyzed using the same protocols. No blinding procedure was introduced, but experimental steps required the very same very well defined steps. Image analysis was performed in the same way for all cell types.

Reporting for specific materials, systems and methods

We require information from authors about some types of materials, experimental systems and methods used in many studies. Here, indicate whether each material, system or method listed is relevant to your study. If you are not sure if a list item applies to your research, read the appropriate section before selecting a response.

Materials & experimental systems

n/a	Involved in the study	
☒	☐	Antibodies
☒	☐	Eukaryotic cell lines
☒	☐	Palaeontology and archaeology
☒	☐	Animals and other organisms
☒	☐	Clinical data
☒	☐	Dual use research of concern
☒	☐	Plants

Methods

n/a	Involved in the study	
☒	☐	ChIP-seq
☐	☒	Flow cytometry
☒	☐	MRI-based neuroimaging

Plants

Seed stocks

Report on the source of all seed stocks or other plant material used. If applicable, state the seed stock centre and catalogue number. If plant specimens were collected from the field, describe the collection location, date and sampling procedures.

Novel plant genotypes

Describe the methods by which all novel plant genotypes were produced. This includes those generated by transgenic approaches, gene editing, chemical/radiation-based mutagenesis and hybridization. For transgenic lines, describe the transformation method, the number of independent lines analyzed and the generation upon which experiments were performed. For gene-edited lines, describe the editor used, the endogenous sequence targeted for editing, the targeting guide RNA sequence (if applicable) and how the editor was applied.

Authentication

Describe any authentication procedures for each seed stock used or novel genotype generated. Describe any experiments used to assess the effect of a mutation and, where applicable, how potential secondary effects (e.g. second site T-DNA insertions, mosiacism, off-target gene editing) were examined.

Flow Cytometry

Plots

Confirm that:

☐ The axis labels state the marker and fluorochrome used (e.g. CD4-FITC).

☐ The axis scales are clearly visible. Include numbers along axes only for bottom left plot of group (a 'group' is an analysis of identical markers).

☐ All plots are contour plots with outliers or pseudocolor plots.

☐ A numerical value for number of cells or percentage (with statistics) is provided.

Methodology

Sample preparation

Cells were grown in the appropriate growth medium to an OD600 circa 0.2 - 0.4 and 500 uL was transferred to a sterile 1.5 mL eppendorf tube. A volume of fixation buffer (PBS with 0.9% NaCl and 0.12% formaldehyde) was added to the cells and gently mixed. To 810 uL of buffered DAPI (0.9% NaCl with 1 ug/mL DAPI), 100 uL of the above cell sample was added. This was incubated on ice for 3 minutes.

Instrument

BD FACSymphony A5 Cell Analyzer

Software

Flow cytometry signals were analyzed in using a customaized Python code.

Cell population abundance

All objects which were DAPI positive were noted as individual cells.

Gating strategy

Instrument was operated with a SSC gain of 1000 and flow rate of 1 uL / s. Signal was collected for 100 seconds per sample. DAPI promoted a distinctly high fluorescence signal of cells. All objects which were DAPI positive were noted as individual cells. We found that applying an automated gating procedure (described in Razo-Mejia et al did not significantly influence our estimate of cells per unit biomass.

☐ Tick this box to confirm that a figure exemplifying the gating strategy is provided in the Supplementary Information.
